# Acute Care Use by Breast Cancer Patients on Adjuvant Chemotherapy in Alberta: Demonstrating the Importance of Measurement to Improving Quality

**DOI:** 10.3390/curroncol28060375

**Published:** 2021-11-02

**Authors:** Che Hsuan David Wu, May Lynn Quan, Shiying Kong, Yuan Xu, Jeffrey Q. Cao, Sasha Lupichuk, Lisa Barbera

**Affiliations:** 1 Department of Oncology, Cumming School of Medicine, University of Calgary, Calgary, AB T2N1N4, Canada; chehsuan@alumni.ubc.ca (C.H.D.W.); maylynn.quan@ahs.ca (M.L.Q.); yuxu@ucalgary.ca (Y.X.); jeffrey.cao@ahs.ca (J.Q.C.); sasha.lupichuk@ahs.ca (S.L.); 2 Tom Baker Cancer Centre, Calgary, AB T2N4N2, Canada; 3 Department of Surgery, Cumming School of Medicine, University of Calgary, Calgary, AB T2N1N4, Canada; 4 Department of Community Health Sciences, Cumming School of Medicine, University of Calgary, Calgary, AB T2N1N4, Canada; 5 Department of Analytics, Alberta Health Services, Calgary, AB T3B0M6, Canada; shiying.kong@ucalgary.ca

**Keywords:** breast cancer, chemotherapy, acute care use, quality, healthcare

## Abstract

Breast cancer patients receiving adjuvant chemotherapy are at increased risk of acute care use. The incidence of emergency department (ED) visits and hospitalizations (H) have been characterized in other provinces but never in Alberta. We conducted a retrospective population-based cohort study using administrative data of women with stage I-III breast cancer receiving adjuvant chemotherapy. Rates of ED and H use in the 180 days following chemotherapy initiation were determined, and logistic regression was performed to identify risk factors. We found that 47% of women receiving adjuvant chemotherapy experienced ED or H, which compared favourably to other provinces. However, Alberta had the highest rate of febrile neutropenia-related ED visits, and among the highest chemotherapy-related ED visits. The incidence of acute care use increased over time, and there were significant institutional differences despite operating under a single provincial healthcare system. Our study demonstrates the need for systematic measurement and the importance of quality improvement programs to address this gap.

## 1. Introduction

Breast cancer is the most common cancer in Canadian women [1], with a significant proportion requiring chemotherapy for high-risk disease including those with node-positive, triple-negative, or HER2-positive cancers. Receipt of adjuvant chemotherapy has been associated with higher incidence of acute care use [2,3], and rates are substantially higher in population data than reported in clinical trials [4].

A large Canadian study [5] evaluated rates of emergency department (ED) and hospitalizations (H) for breast cancer patients receiving adjuvant chemotherapy in four Canadian provinces: British Columbia, Manitoba, Ontario, and Nova Scotia. Alberta was not included, and these metrics have never been reported for this province.

The purpose of this study was to describe the frequency of ED and H for breast cancer patients receiving adjuvant chemotherapy in Alberta, and describe factors associated with these outcomes.

## 2. Materials and Methods

### 2.1. Study Design and Cohort Creation

This is a retrospective population-based cohort study using observational data of women with breast cancer. The study was set in the province of Alberta, Canada, with a total population of 4.4 million people centred primarily around 2 large urban centres. All care is provided by a publicly funded single-payer health plan.

We included all adult women with newly diagnosed stage I-III invasive breast cancer between January 2004 and December 2015, who were treated with ≥1 cycle of adjuvant chemotherapy within 6 months of curative-intent surgery. Patients were excluded if chemotherapy was given in the neoadjuvant setting, or if there was a diagnosis of multiple breast primaries or prior cancers. Those with missing identifier data or an invalid health card number were excluded from the statistical analysis. Ethics approval was obtained from the Health Research Ethics Board of Alberta Cancer Committee (HREBA.CC-18-0166; first approved 19 July 2018, last renewed 17 June 2021).

### 2.2. Data Sources and Covariates

Several sources of administrative health care data were linked deterministically using unique encoded identifiers. Cancer diagnoses and chemotherapy administration were obtained from the Alberta Cancer Registry (ACR). The ACR captures all incident cases of cancer via a system of mandatory reporting. This includes demographic information, cancer staging, and treatment-related details including date of chemotherapy and type of regimen. The ACR has been awarded gold level certification by the North American Association of Central Cancer Registries for data quality and completeness [6]. ED visits are captured in the Canadian Institute for Health Information (CIHI) National Ambulatory Care Reporting System (NACRS) [7], which contains data for all hospital-based and community-based ambulatory care. Data collected include visit date, visit type (planned vs. unplanned), visit reasons, and triage level. ED visit reasons are coded using the Canadian Emergency Department Diagnoses Shortlist, which are mapped to ICD-10-CA codes. Visit triage levels are recorded by the Canadian Triage and Acuity Scale (CTAS), ranging from level 1 (most urgent, requiring resuscitation) to level 5 (chronic, non-urgent conditions). Hospitalizations are captured in the CIHI Discharge Abstract Database (DAD) [8], which includes demographic, administrative, and clinical data from all hospital discharges. This includes the responsible diagnosis and any secondary diagnoses related to the admission. Geographic region of residence, neighborhood income, and neighborhood education levels were obtained from the 2011 Canadian Census program [9] via postal code linkage [10]. Tumour stage was obtained from the Collaborative Staging Database [6]. Comorbidity was calculated using the Deyo modification of the Charlson comorbidity index (CCI) based on diagnoses coded in CIHI DAD in the 5 years prior to diagnosis, with scores for primary cancer subtracted [11].

### 2.3. Outcome Definitions

The primary outcome was unplanned acute care use during the observation window, which we defined as the period of 180 days following the first chemotherapy administration to allow for comparison with other published work in this area [5]. Patient-specific windows, where the observation period ended 30 days following last chemotherapy administration, provided similar results and are not reported. Unplanned acute care use included ED visits only (ED), ED visits that lead to hospitalization (ED→H), and direct hospitalizations (H). The latter group comprised a small percentage of patients (<2%); we therefore focused the remainder of our analysis on ED and ED→H.

Visit reasons were classified as chemotherapy-related using a priori knowledge of chemotherapy toxicities, independent review of ICD-10-CA codes, and the validated algorithm originally developed by Hassett et al. [2] Rates of febrile neutropenia (FN) were captured according to a previously validated algorithm [12] that defines FN-related visits as those with ‘neutropenia’ as the main diagnostic code.

### 2.4. Statistical Analysis

Descriptive analyses were performed to describe the cohort, with the Wilcoxon rank sum test for continuous variables and the chi-square test for proportions. Univariable and multivariable logistic regression models were used to identify variables associated with each of the acute care outcomes. Covariates include age, CCI score, rural versus urban residence (<30k population vs. ≥30k), neighbourhood income and education quintiles, year of diagnosis, overall stage, biomarker profile (estrogen receptor (ER), progesterone receptor (PR), HER2), type of breast surgery (mastectomy vs. breast conservation), radiotherapy use, chemotherapy regimen (taxane/non-anthracycline, anthracycline/non-taxane, anthracycline/taxane, or other), G-CSF use, and cancer centre. All statistical analyses were completed using SAS version 14.2 (SAS Institute, Cary, NC, USA).

## 3. Results

We identified 6336 women who met our inclusion criteria. Table 1 describes the baseline patient characteristics. Median age was 52 years, with majority having a CCI score of 0 (73%) and stage II disease (59%). Thirty-nine percent of patients had tumours that were ER or PR positive and HER2 negative. As HER2 status was not reported to the ACR until 2010, 40% of patients had an incomplete biomarker profile. Rates of mastectomy (52%) versus breast conservation (48%) were similar. The majority received a taxane/non-anthracycline regimen (39%) or an anthracycline/taxane regimen (39%). Amongst patients who received a taxane-containing regimen, docetaxel was used in the majority (95%). Approximately 40% received G-CSF. The vast majority (90%) received chemotherapy at one of our two tertiary cancer centres. Patients were unevenly distributed across income and education quintiles, with more patients living in wealthier and more educated neighbourhoods.

**Figure 1:**
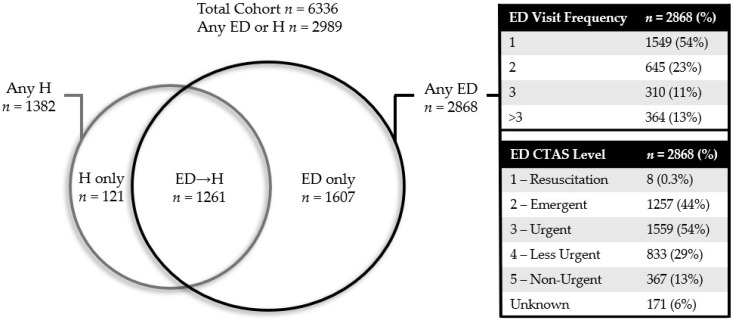
Distribution of acute care utilization in our cohort. Patients with any ED use were further categorized by visit frequency and Canadian Triage and Acuity Scale (CTAS). As patients may have multiple visits, the total percentage for CTAS exceeds 100%.

Of our entire cohort, there were 2989/6336 unique patients (47%) who experienced ED or H. As shown in Figure 1, this consisted of patients who experienced ED only (25%, 1607/6336), ED→H (20%, 1261/6336), or H only (2%, 121/6336). This latter category was excluded from further analysis. The proportion of patients who required admission after presenting to the ED was 44% (1261/2868). Among the patients who accessed the ED, most (54%, 1549/2868) had a single ED visit only, and the majority (54%, 1559/2868) had at least one urgent ED visit (CTAS level 1–3). There was a higher proportion of CTAS 1–3 visits among patients with ED→H (59%, 749/1261) than ED alone (50%, 810/1607). We identified 2071 patients with at least one chemotherapy-related ED visit, which constituted 34% of all eligible patients and 72% of those with any ED visits. There were 815 patients with at least one FN-related ED visit, which constituted 13% of all eligible patients and 28% of those with any ED visits.

**Figure 2:**
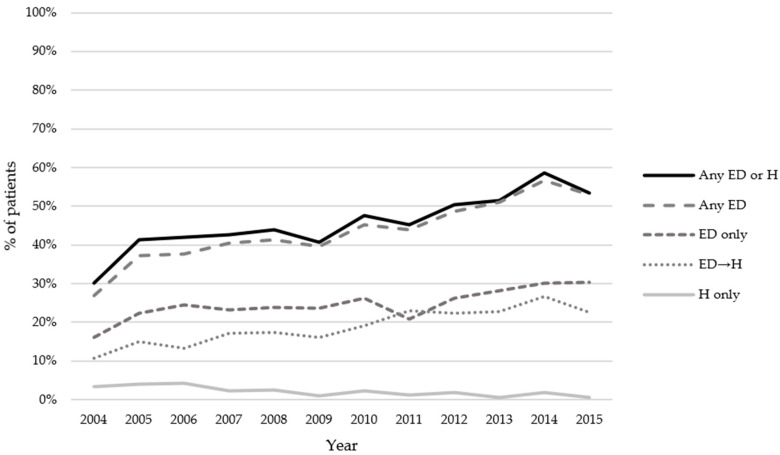
Percentage of patients experiencing acute care use in the year they received chemotherapy.

Our cohort experienced 6432 ED visits during the observation period, with the vast majority being unplanned (97%). The 197 (3%) planned ED visits were excluded from further analysis. Approximately 26% of ED visits were emergent or resuscitation (CTAS 1–2), 35% were urgent (CTAS 3), and 33% of ED visits were less urgent or non-urgent (CTAS 4–5). Chemotherapy-related visits comprised 54% of visits. Timing of ED visits consisted of 33% occurring during weekdays between 8 a.m.–4 p.m., 30% between 4 p.m.–12 a.m., and 8% between 12 a.m.–8 a.m. The respective frequencies on weekends were 15%, 12%, and 3%. ED visits increased over the years, with approximately 30% in 2004 versus over 50% in 2015 (Figure 2). The proportion of patients experiencing ED only and ED→H in the year they received chemotherapy have both increased over time.

### Logistic Regression Models for ED and ED→H

Table 2 and Table 3 present univariable and multivariable results for models evaluating those with ED visits only and those with ED→H, respectively.

On multivariate analysis, later year of diagnosis was associated with higher ED-only use (OR 1.06, 95% CI 1.02–1.1). Factors significantly associated with lower ED-only use included urban residence (OR 0.49, 95% CI); higher education (fourth quintile OR 0.78, 95% CI 0.63–0.97); and chemotherapy administration at Urban Centre #2 (OR 0.71, 95% CI 0.62–0.81) or at multiple facilities (OR 0.69, 95% CI 0.48–0.99).

Factors significantly associated with higher ED→H use on multivariate analysis were CCI scores of 1 (OR 1.26, 95% CI 1.07–1.47), 2 (OR 1.65, 95% CI 1.21–2.24), and >2 (OR 2.32, 95% CI 1.55–3.48); biomarker profile ER+ or PR+ and HER2+ (OR 1.8, 95% CI 1.46–2.22), and ER- PR- HER2+ (OR 2.1, 95% CI 1.48–2.98); and use of G-CSF (OR 3.96, 95% CI 3.39–4.62). Factors significantly associated with lower ED→H use include higher income (fourth quintile OR 0.74, 95% CI 0.58–0.94; fifth quintile OR 0.74, 95% CI 0.58–0.95); anthracycline/taxane chemotherapy (OR 0.53, 95% CI 0.3–0.94); and chemotherapy administration at Urban Centre #2 (OR 0.56, 95% CI 0.48–0.65).

## 4. Discussion

We found that 47% of women with stage I–III breast cancer experienced unplanned acute care use within 180 days after initiating adjuvant chemotherapy. Over 30% of eligible study patients had a chemotherapy-related ED visit, and 13% had a FN-related ED visit. Over half of patients had urgent ED visits, and two-thirds of these visits occurred during the business week and prior to midnight. The rates of acute care use have steadily increased over time, from 30% in 2004 to over 50% in 2015. Although studies have reported on variations in practice and outcomes in Alberta for other conditions and treatments [13,14,15,16], this is the first time these specific observations have been made in this province.

A large Canadian study investigating this patient group across four provinces was conducted in 2019 [5], which served as a reference comparator for our study. Our overall patient population was younger (median age 52 vs. 60–61 years) but was similar in terms of stage and receptor status. The rate of ED-only use in Alberta was 25%, which is lower than Ontario (36%) and Nova Scotia (30%), similar to Manitoba (24%), and higher than British Columbia (16%). ED→H rates were highest in Alberta at 20% compared to the other provinces (14–17%). H was lowest in Alberta at 2% of eligible patients versus 6–27% elsewhere. Overall, the proportion of any ED or H use in Alberta is 47%, which is favourable compared to Nova Scotia (65%), British Columbia (60%), and Ontario (58%), and similar to Manitoba (44%). However, Alberta had the highest proportion of patients with a FN-related ED visit at 13% (compared to 9–11%). The proportion of chemotherapy-related ED visits was also among the highest at 33% (compared to 25–35%). Our rates of any ED or H use are similar to another Ontario study [17] reporting a rate of 43% within 30 days of last chemotherapy administration. The timing of ED visits is also similar to Ontario [18], which reported 71% of visits occurred outside of regular business hours. However, the hospitalization rate after presenting to ED was higher in Alberta (44% vs. 34%) [18]. Our study further adds to the similarities and differences in acute care use between Canadian provinces and highlight areas for targeted improvement, particularly in the management and prevention of FN- and chemotherapy-related ED visits.

Significant factors on multivariable analysis for increased acute care use were similar to those previously reported [17], including greater comorbidities, lower education levels, and rural geographic areas. In our study, Urban Centre #2 showed lower rates of both ED and ED→H use compared to Urban Centre #1 and community centres. The degree of regional and cancer-centre variability was not expected since Alberta operates under a unified provincial healthcare system. While the provincial health agency strives to standardize practice and ensure high quality care is delivered regardless of location, known variations exist. Our results suggest room for improvement. Examining factors such as medical oncology prescribing and on-call practices, nursing care models, and pharmacy education services may identify differences that can be leveraged to have an impact.

Additional variables associated with higher rates of acute care use included later year of diagnosis. This may relate to evolving chemotherapy prescribing practices, or a reflection of limited cancer centre-based resources in the setting of a growing, more complex patient population. HER2 positive tumour status was significant for more ED→H. This may be related to higher toxicities with the addition of anti-HER2 therapy, including risk of left ventricular dysfunction. G-CSF use was significantly associated with higher rates of ED→H. As the temporal relationship between G-CSF use and acute care visits is not known, this may be explained by secondary use after an FN event or G-CSF side effects such as leukocytosis and severe ostealgia. Given the limitations of our dataset, the large proportion of patients receiving G-CSF, and the strong association between G-CSF use and ED→H, further study into this area is warranted.

A SEER-Medicare database study showed that combination anthracycline/docetaxel regimens were associated with the highest rates of hospitalizations [19]. Data from Ontario has also found that use of docetaxel was associated with increased acute care use [17]. Although almost 95% of taxane-including regimens in our study used docetaxel, we did not find that anthracycline/taxane or taxane regimens were associated with increased acute care use in comparison to non-anthracycline/non-taxane regimens. Anthracycline/taxane regimens were associated with less ED→H in comparison to non-anthracycline/non-taxane regimens in our population. This may be explained by the small number of patients in the referent non-anthracycline/non-taxane regimen group, and the tendency to avoid anthracyclines and taxanes in patients with co-morbidities.

The benchmark for all-cause any ED or H use is 32.5% according to a previous study [20]. Alberta had a range of 38–73% across institutions, with no institution achieving the benchmark. Our results demonstrate an opportunity to improve our acute care utilization in this population.

Our study has inherent limitations associated with administrative and registry data including its retrospective nature, potential for missing data or coding errors, and lack of patient-specific and visit-specific context. We do not have data on endocrine therapy use available in our database. Endocrine therapy initiation likely occurred within the 180-day window for most patients with HR+ tumours who finished chemotherapy. Serious adverse events such as venous thromboembolism from tamoxifen may have contributed to acute care visits. Finally, we have only studied patients who received adjuvant chemotherapy. With the shift to increasing prescription of neoadjuvant chemotherapy for HER2+ and triple negative tumours, study of acute care use in the pre-operative period is warranted.

## 5. Conclusions

Acute care use in early-stage breast cancer patients receiving adjuvant chemotherapy in Alberta has steadily risen over the years. Overall rates of ED or H use are similar if not favourable compared to other provinces. However, rates of chemotherapy-related and FN-related visits are among the highest. There are substantial institutional variations in acute care use, despite having a single provincial healthcare agency. In the years of this cohort, the Alberta provincial cancer agency did not have a strong quality mandate and issues such as chemotherapy-related toxicity were not explored. In provinces with a strong programmatic emphasis on quality, robust programs can be created and possible solutions investigated. For example, a cluster randomized trial [21] investigated two models of toxicity management in Ontario. This difference in provincial approaches highlights the potential quality gaps that can develop or exist in the absence of systematic measurement and underscores the need for robust quality programs within cancer agencies. The advent of a new Quality Safety Committee within the provincial cancer agency is expected to increase the emphasis on quality and, over time, narrow this measurement gap.

## Figures and Tables

**Table 1:** Study cohort demographic data. IQR: interquartile range.

Variable		Entire Cohort	ED only	ED→H
		N = 6336	N = 1607	N = 1261
Age	Median (IQR)	52 (45–60)	52 (45–60)	53 (46–61)
CCI Score	0	4637 (73%)	1137 (71%)	853 (68%)
	1	1327 (21%)	361 (23%)	293 (23%)
	2	247 (4%)	72 (5%)	72 (6%)
	>2	125 (2%)	37 (2%)	43 (3%)
Urban	Rural	1186 (19%)	461 (29%)	275 (22%)
	Urban	5148 (81%)	1146 (71%)	985 (78%)
Quintile Income	1 (low)	783 (12%)	210 (13%)	183 (15%)
	2	1203 (19%)	325 (20%)	269 (21%)
	3	1163 (18%)	313 (20%)	236 (19%)
	4	1513 (24%)	371 (23%)	280 (22%)
	5	1672 (26%)	388 (24%)	292 (23%)
Quintile Education	1 (low)	944 (15%)	297 (19%)	225 (18%)
	2	1181 (18%)	343 (21%)	242 (19%)
	3	1280 (20%)	313 (20%)	253 (20%)
	4	1671 (26%)	354 (22%)	314 (25%)
	5	1258 (20%)	300 (19%)	226 (18%)
Year of Diagnosis	2004	242 (4%)	39 (2%)	26 (2%)
	2005	368 (6%)	82 (5%)	55 (4%)
	2006	393 (6%)	96 (6%)	52 (4%)
	2007	483 (8%)	112 (7%)	83 (7%)
	2008	464 (7%)	111 (7%)	81 (6%)
	2009	557 (9%)	132 (8%)	89 (7%)
	2010	570 (9%)	149 (9%)	109 (9%)
	2011	614 (10%)	128 (8%)	141 (11%)
	2012	688 (11%)	180 (11%)	154 (12%)
	2013	648 (10%)	182 (11%)	148 (12%)
	2014	675 (11%)	203 (13%)	180 (14%)
	2015	634 (10%)	193 (12%)	143 (11%)
Stage	I	1478 (23%)	345 (22%)	274 (22%)
	II	3715 (59%)	972 (61%)	738 (59%)
	III	1143 (18%)	290 (18%)	249 (20%)
T Stage	T1	2745 (43%)	714 (44%)	529 (42%)
	T2	3141 (50%)	788 (49%)	638 (51%)
	T3	398 (6%)	91 (6%)	84 (7%)
	T4	46 (1%)	12 (1%)	9 (1%)
N Stage	N0	2949 (47%)	683 (43%)	559 (44%)
	N1	2399 (38%)	670 (42%)	489 (39%)
	N2	645 (10%)	176 (11%)	133 (11%)
	N3	343 (5%)	78 (5%)	80 (6%)
Biomarker Profile	ER+ or PR+ HER2+	658 (10%)	150 (9%)	204 (16%)
	ER+ or PR+ HER2-	2445 (39%)	690 (43%)	490 (39%)
	ER- and PR- and HER2+	180 (3%)	42 (3%)	60 (5%)
	ER- and PR- and HER2-	515 (8%)	149 (9%)	113 (9%)
	Unknown	2538 (40%)	576 (36%)	394 (31%)
Surgery	Lumpectomy	3014 (48%)	744 (46%)	571 (45%)
	Mastectomy	3322 (52%)	863 (54%)	690 (55%)
Radiation	No	1898 (30%)	464 (29%)	379 (30%)
	Yes	4438 (70%)	1143 (71%)	882 (70%)
Regimen Group	Anthracycline/ Non-Taxane	1155 (18%)	230 (14%)	143 (11%)
	Taxane/ Non-Anthracycline	2638 (42%)	641 (40%)	614 (49%)
	Anthracycline/Taxane	2456 (39%)	713 (44%)	486 (39%)
	Other	87 (1%)	23 (1%)	18 (1%)
G-CSF Cycles Received	0	3914 (62%)	970 (60%)	516 (41%)
	1	315 (5%)	81 (5%)	121 (10%)
	2	350 (6%)	92 (6%)	137 (11%)
	3	764 (12%)	194 (12%)	210 (17%)
	>3	993 (16%)	270 (17%)	277 (22%)
Cancer Centre	Community #1	210 (3%)	69 (4%)	69 (6%)
	Community #2	44 (1%)	18 (1%)	14 (1%)
	Community #3	143 (2%)	58 (4%)	33 (3%)
	Community #4	66 (1%)	25 (2%)	8 (1%)
	Urban #1	2962 (47%)	819 (51%)	673 (53%)
	Urban #2	2739 (43%)	562 (35%)	417 (33%)
	Multiple	168 (3%)	53 (3%)	46 (4%)

**Table 2:** Univariable and multivariable logistic regression models for ED only. Statistically significant values are bolded. OR: odds ratio; CI: confidence interval; CCI: Charlson Comorbidity index; G-CSF: granulocyte-colony stimulating factor.

Variable		Univariable OR (95% CI)	Multivariable OR (95% CI)
Age	Continuous	1 (0.99 to 1)	0.99 (0.99 to 1)
CCI Score	0	Reference	Reference
	1	1.15 (1 to 1.32)	1.12 (0.97 to 1.29)
	2	1.28 (0.97 to 1.7)	1.19 (0.89 to 1.6)
	>2	1.29 (0.88 to 1.91)	1.17 (0.78 to 1.75)
Urban	Rural	Reference	Reference
	Urban	**0.45 (0.39 to 0.51)**	**0.49 (0.42 to 0.58)**
Quintile Income	1 (low)	Reference	Reference
	2	1.01 (0.82 to 1.24)	1.01 (0.82 to 1.24)
	3	1 (0.82 to 1.23)	1.1 (0.89 to 1.37)
	4	0.89 (0.73 to 1.08)	1.08 (0.87 to 1.34)
	5	0.82 (0.68 to 1)	1.14 (0.91 to 1.43)
Quintile Education	1 (low)	Reference	Reference
	2	0.89 (0.74 to 1.07)	1.02 (0.84 to 1.24)
	3	**0.7 (0.58 to 0.85)**	0.87 (0.71 to 1.07)
	4	**0.59 (0.49 to 0.7)**	**0.78 (0.63 to 0.97)**
	5	**0.68 (0.56 to 0.82)**	0.99 (0.79 to 1.24)
Year of Diagnosis	Continuous	**1.05 (1.03 to 1.07)**	**1.06 (1.02 to 1.1)**
Stage	I	Reference	Reference
	II	**1.16 (1.01 to 1.34)**	1.06 (0.91 to 1.24)
	III	1.12 (0.93 to 1.34)	0.95 (0.76 to 1.18)
Biomarker Profile	ER+ or PR+ HER2-	Reference	Reference
	ER+ or PR+ HER2+	**0.75 (0.61 to 0.92)**	0.88 (0.71 to 1.09)
	ER- and PR- and HER2+	0.77 (0.54 to 1.1)	0.89 (0.62 to 1.29)
	ER- and PR- and HER2-	1.04 (0.84 to 1.28)	1.06 (0.85 to 1.31)
	Other	**0.75 (0.66 to 0.85)**	1.09 (0.87 to 1.37)
Surgery	Lumpectomy	Reference	Reference
	Mastectomy	1.07 (0.96 to 1.2)	1.1 (0.95 to 1.27)
Radiation	No	Reference	Reference
	Yes	1.07 (0.95 to 1.21)	1.05 (0.89 to 1.23)
Regimen Group	Neither	Reference	Reference
	Anthracycline only	0.68 (0.41 to 1.12)	0.88 (0.52 to 1.46)
	Both	1.12 (0.69 to 1.82)	1.15 (0.69 to 1.91)
	Taxane only	0.88 (0.54 to 1.43)	0.83 (0.5 to 1.37)
G-CSF Received	No	Reference	Reference
	Yes	1.1 (0.98 to 1.24)	0.88 (0.77 to 1.01)
Cancer Centre	Urban #1	Reference	Reference
	Urban #2	**0.67 (0.6 to 0.76)**	**0.71 (0.62 to 0.81)**
	Community	**1.54 (1.25 to 1.89)**	1.12 (0.89 to 1.41)
	Multiple	1.2 (0.86 to 1.68)	**0.69 (0.48 to 0.99)**

**Table 3:** Univariable and multivariable logistic regression models for ED→H. Statistically significant values are bolded. OR: odds ratio; CI: confidence interval; CCI: Charlson Comorbidity index; G-CSF: granulocyte-colony stimulating factor.

Variable		Univariable OR (95% CI)	Multivariable OR (95% CI)
Age	Continuous	1.01 (1 to 1.01)	1.01 (1 to 1.01)
CCI Score	0	Reference	Reference
	1	**1.26 (1.08 to 1.46)**	**1.26 (1.07 to 1.47)**
	2	**1.78 (1.33 to 2.37)**	**1.65 (1.21 to 2.24)**
	>2	**2.33 (1.6 to 3.39)**	**2.32 (1.55 to 3.48)**
Urban	Rural	Reference	Reference
	Urban	**0.79 (0.67 to 0.91)**	0.97 (0.81 to 1.16)
Quintile Income	1 (low)	Reference	Reference
	2	0.95 (0.76 to 1.17)	0.92 (0.73 to 1.16)
	3	0.84 (0.67 to 1.04)	0.84 (0.66 to 1.07)
	4	**0.75 (0.6 to 0.92)**	**0.74 (0.58 to 0.94)**
	5	**0.7 (0.57 to 0.86)**	**0.74 (0.58 to 0.95)**
Quintile Education	1 (low)	Reference	Reference
	2	0.82 (0.67 to 1.01)	0.85 (0.68 to 1.06)
	3	**0.79 (0.65 to 0.97)**	0.88 (0.7 to 1.12)
	4	**0.74 (0.61 to 0.9)**	0.91 (0.71 to 1.15)
	5	**0.7 (0.57 to 0.87)**	0.91 (0.7 to 1.18)
Year of Diagnosis	Continuous	**1.08 (1.06 to 1.1)**	0.97 (0.93 to 1.01)
Stage	I	Reference	Reference
	II	1.08 (0.93 to 1.26)	1.08 (0.91 to 1.29)
	III	**1.22 (1.01 to 1.48)**	1.24 (0.97 to 1.58)
Biomarker Profile	ER+ or PR+ HER2-	Reference	Reference
	ER+ or PR+ HER2+	**1.8 (1.48 to 2.18)**	**1.8 (1.46 to 2.22)**
	ER- and PR- and HER2+	**2 (1.44 to 2.77)**	**2.1 (1.48 to 2.98)**
	ER- and PR- and HER2-	1.12 (0.89 to 1.41)	1.17 (0.91 to 1.49)
	Other	**0.73 (0.63 to 0.85)**	1.02 (0.78 to 1.31)
Surgery	Lumpectomy	Reference	Reference
	Mastectomy	1.12 (0.99 to 1.27)	1.06 (0.91 to 1.25)
Radiation	No	Reference	Reference
	Yes	0.99 (0.87 to 1.14)	0.92 (0.77 to 1.1)
Regimen Group	Neither	Reference	Reference
	Anthracycline only	0.57 (0.33 to 1)	0.69 (0.39 to 1.22)
	Both	1 (0.58 to 1.71)	0.53 (0.3 to 0.94)
	Taxane only	1.23 (0.72 to 2.11)	0.87 (0.5 to 1.52)
G-CSF Received	No	Reference	Reference
	Yes	**3.06 (2.69 to 3.47)**	**3.96 (3.39 to 4.62)**
Cancer Centre	Urban #1	Reference	Reference
	Urban #2	**0.61 (0.53 to 0.7)**	**0.56 (0.48 to 0.65)**
	Community	1.25 (1 to 1.56)	0.79 (0.61 to 1.02)
	Multiple	1.29 (0.91 to 1.83)	1.07 (0.73 to 1.58)

## Data Availability

No new data were created or analyzed in this study. Data sharing is not applicable to this article.
